# Chloroquine alleviates etoposide-induced centrosome amplification by inhibiting CDK2 in adrenocortical tumor cells

**DOI:** 10.1038/oncsis.2015.37

**Published:** 2015-12-21

**Authors:** T-Y Chen, J-S Syu, T-C Lin, H-l Cheng, F-l Lu, C-Y Wang

**Affiliations:** 1 Department of Cell Biology and Anatomy, College of Medicine, National Cheng Kung University, Tainan, Taiwan; 2 Institute of Basic Medical Sciences, College of Medicine, National Cheng Kung University, Tainan, Taiwan; 3 Institute of Biotechnology, College of Bioscience and Biotechnology, National Cheng Kung University, Tainan, Taiwan; 4 Department of Biotechnology and Bioindustry Sciences, College of Bioscience and Biotechnology, National Cheng Kung University, Tainan, Taiwan

## Abstract

The antitumor drug etoposide (ETO) is widely used in treating several cancers, including adrenocortical tumor (ACT). However, when used at sublethal doses, tumor cells still survive and are more susceptible to the recurring tumor due to centrosome amplification. Here, we checked the effect of sublethal dose of ETO in ACT cells. Sublethal dose of ETO treatment did not induce cell death but arrested the ACT cells in G2/M phase. This resulted in centrosome amplification and aberrant mitotic spindle formation leading to genomic instability and cellular senescence. Under such conditions, Chk2, cyclin A/CDK2 and ERK1/2 were aberrantly activated. Pharmacological inactivation of Chk2, CDK2 or ERK1/2 or depletion of CDK2 or Chk2 inhibited the centrosome amplification in ETO-treated ACT cells. In addition, autophagy was activated by ETO and was required for ACT cell survival. Chloroquine, the autophagy inhibitor, reduced ACT cell growth and inhibited ETO-induced centrosome amplification. Chloroquine alleviated CDK2 and ERK, but not Chk2, activation and thus inhibited centrosome amplification in either ETO- or hydroxyurea-treated ACT cells. In addition, chloroquine also inhibited centrosome amplification in osteosarcoma U2OS cell lines when treated with ETO or hydroxyurea. In summary, we have demonstrated that chloroquine inhibited ACT cell growth and alleviated DNA damage-induced centrosome amplification by inhibiting CDK2 and ERK activity, thus preventing genomic instability and recurrence of ACT.

## Introduction

Adrenal gland, which is composed of the cortex and medulla, is the most important endocrine organ that lies on top of the kidney. Adrenocortex is the major site of steroidogenesis in response to adrenocorticotropic hormone stimulation, and its abnormal growth leads to adrenocortical tumor (ACT).^1^ ACT is a rare but aggressive cancer that occurs in either adult or children. Correlated with its physiological function, tumor that occurs in the adrenocortex shows many hormonal symptoms that are similar to those seen in patients who suffer from steroid hormone excess, such as Cushing's syndrome and virilization, exhibiting high levels of cortisol and androgen, respectively.^2, 3^ The pathogenesis of ACT is not completely understood; overexpression of insulin-like growth factor 2 and steroidogenic factor 1 are involved in the development of ACT.^4, 5, 6, 7, 8^ Constitutive activated Wnt/beta-catenin signaling is also observed in ACT patents.^9, 10^ Owing to its complexity and poor prognosis, the treatment of ACT mainly depends on surgical resection and cytotoxic therapies, such as etoposide (ETO), doxorubicin, cisplatin and mitotane treatment.^11^ Among these drugs, ETO is one of the most commonly used antitumor drugs in the world.

ETO (VP-16) is a widely used anticancer drug in clinic. It is a topoisomerase II inhibitor that induces DNA double-strand breaks followed by cell cycle arrest or apoptosis.^12^ As treatment of ETO induces DNA double-strand breaks, DNA damage response is triggered and several damage markers can be observed including γ-H2AX, phosphorylation and accumulation of p53.^13, 14^ This drug has been used for treating adrenal cortical carcinoma for long,^15^ however, the molecular mechanism by which ETO affects ACT is still unclear.

When subtoxic doses of cytotoxic drug are administered, some tumor cells still survive and become more malignant owing to genomic instability, thus promoting recurring tumor.^16, 17^ When exposed to sublethal dose of chemotherapy, tumor cells undergo cell cycle arrest and centrosome amplification.^18, 19^ Thus, when patient case from the chemotherapy, these tumor cells containing multiple centrosomes re-enter into cell cycle and form multiple mitotic spindle poles with misalignment of chromosomes during mitosis.^17^ Errors in mitosis lead to enlarged nucleus, micronuclei or even cytokinesis failure; these are all hallmarks of genomic instability.^17, 20, 21^ Thus, precise control of centrosome homeostasis is important for the maintenance of genomic integrity. When cells harbor supernumerary centrosomes and there is deficiency in DNA repair machinery, these cells are more susceptible to malignancy.^22^

The centrosome consists of a pair of centrioles and the surrounding pericentriolar material. It is the major microtubule nucleating site; this microtubule nucleation activity orchestrates cytoskeleton during interphase and mitotic spindle at M phase.^23^ Centrosome duplication coordinates with DNA replication.^24^ During the S phase, activated CDK2 triggers DNA replication and centrosome duplication simultaneously. Each centriole serves as a platform for a new centriole to grow in the orthogonal relationship. At G2/M transition, duplicated centrioles separate to the opposite of the nucleus followed by alignment of the chromosomes in the equatorial plate for proper segregation. Thus, the centrosome is required for proper cell cycle progression and depletion of centrosomal proteins leads to cell cycle arrest.^25, 26^ In addition, overexpression of Cyclin A and aberrant activation of CDK2 induces centrosome amplification due to centrosome over-duplication. Thus, precise control of the activity of CDK2 is important to maintain centrosome copy numbers.

Autophagy is a lysosomal-degradation process whereby cells degrade and reutilize the old organelles and proteins to maintain metabolic homeostasis.^27^ Several cellular stresses, such as starvation, DNA damage or hypoxia, increase the activity of autophagy. Unc-51-like kinase 1 (ULK1) and -2 (ULK2), which are the mammalian homologs of Atg1, are required for the induction of autophagy. In the nutrient-enriched environment, ULK1/2 are inhibited by mammalian target of rapammycin. Upon serum starvation, mammalian target of rapammycin is inactivated and thus ULK1/2 phosphorylate mammalian Atg13, focal adhesion kinase family interacting protein of 200 kDa (FIP200) and itself to initiate autophagy.^28^ Two ubiquitination-like processes are required for the autophagosome formation. Atg12, the ubiquitin-like Atg protein, is activated by Atg7, an E1-like enzyme, and Atg10 then conjugated to Atg5 and promotes the formation of the autophagosome precursor.^29^ The other ubiquitin-like protein is microtubule-associated protein l light chain 3 (LC3). LC3 is cleaved and activated by Atg4 to expose the Gly116 residue (LC3-I) followed by conjugation with Atg7. LC3-I is then transferred to Atg3, the E2-like enzyme, through a thioester bond. In the final step, Gly116 residue of LC3-I is conjugated to phosphatidylethanolamine through an amide bond (LC3-II); LC3-II exists in a tight membrane-associated form and promotes the formation of autophagosomes.^30^ Once the autophagosome is formed, it will further fuse with lysosome for degrading the cytoplasmic organelles.

Chloroquine, an anti-malarial drug, is an autophagic inhibitor that acts by preventing autophagosome fusion with lysosome and inhibiting lysosomal acidification.^31^ It has been shown that autophagy protects cancer cells from apoptosis when cells treated with anticancer drugs and thus co-treatment of chloroquine with anticancer drugs accelerates cell death in several cancers such as brain tumor and leukemia.^32, 33, 34, 35, 36^ In this study, we found that chloroquine alleviated ETO- or hydroxyurea-induced centrosome amplification in ACT and osteosarcoma cells. Sublethal dose of ETO treatment induced centrosome amplification followed by aberrant mitosis that led to genomic instability and senescence in ACT cells. ETO-induced Chk2, CDK2 and ERK activation facilitated centrosome amplification. Chloroquine treatment reduced ACT cell growth and inhibited CDK2 and ERK, but not Chk2, signaling and thus prevented centrosome amplification in ETO- or hydroxyurea-treated ACT or osteosarcoma cells. Thus, our study demonstrated the novel function of chloroquine in preventing centrosome amplification upon sublethal dose of ETO or hydroxyurea treatment.

## Results

### ETO inhibits ACT growth

**Figure 1:**
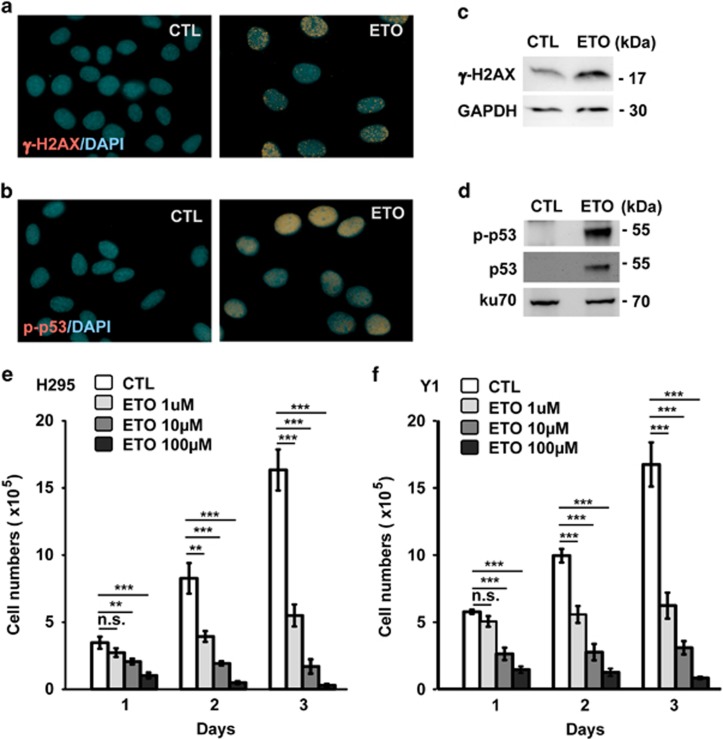
ETO inhibits ACT cell growth. (**a**–**d**) Treatment of ETO (10 μM) for 24 h induces DNA damage response. ETO-treated H295 cells were co-stained with DNA dye (DAPI) with antibodies against (**a**) phospho-Ser139 of H2AX (γ-H2AX) or (**b**) phospho-Ser15 of p53 (p-p53). CTL: control (DMSO) treatment. Extracts of ETO-treated H295 cells were analyzed with antibodies against (**c**) phospho-Ser139 of H2AX (γ-H2AX) and GAPDH, or (**d**) p53, phospho-Ser15 of p53 (p-p53) and Ku70. (**e** and **f**) ETO inhibits ACT cell growth. The numbers of ACT H295 (**e**) and Y1 (**f**) cells were quantified in scramble control (CTL) or ETO treatment at different concentrations for different time points. n.s., no significance, **P<0.01, ***P<0.001.

ETO affects cell cycle progression by inducing DNA damage responses.^14^ To check the effect of ETO on ACT cells, H295 cells, the human ACT cells, were treated with ETO and the phosphorylation of H2AX (γ-H2AX) and p53, the markers of damage responses,^37, 38^ were checked. Following 10 μM ETO treatment for 24 h, the amounts of phosphorylated and total p53 and γ-H2AX were increased dramatically indicating that ETO-induced DNA damages in ACT cells (Figures 1a and d). Then, the growth of ACT cells was checked. H295 and Y1 (mouse ACT) cell lines were treated with ETO at the concentration of 1, 10 and 100 μM. ETO treatment inhibited H295 and Y1 cell growth in time- and dose-dependent manners (Figures 1e and f). Treatment of cells with 1 or 10 μM ETO for 3 days did not induce apoptosis in ACT cells. However, treatment of cells with 100 μM ETO for 3 days induced apoptosis dramatically (data not shown). Thus, we test the effect of sublethal dose of ETO by treating cells with ETO at 10 μM concentration.

### Sublethal dose of ETO treatment leads to polyploidy and cellular senescence

**Figure 2:**
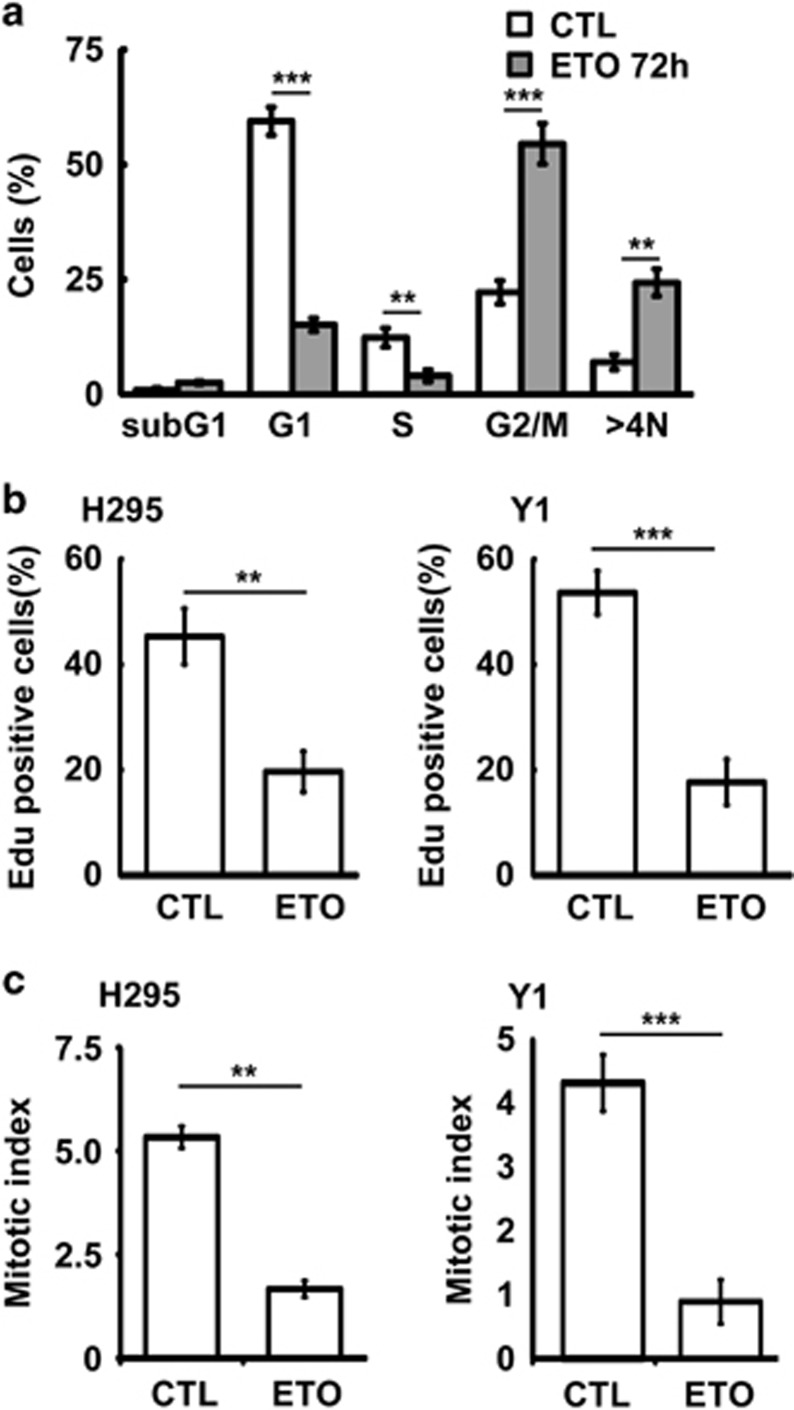
Sublethal dose of ETO treatment disturbs cell cycle profile. (**a**) ETO treatment (10 μM, 72 h) disturbs the cell cycle profile. Quantification of different cell cycle stages in adrenocortical Y1 tumor cells in the presence or absence of ETO. (**b** and **c**) EdU incorporation and mitotic index are reduced in ETO-treated (10 μM, 24 h) adrenocortical H295 and Y1 tumor cell lines. Quantification of EdU incorporation (**b**) or mitotic index (**c**) in scramble control (CTL) or ETO-treated H295 and Y1 cell lines. These results are mean +/−s.d. from three independent experiments; more than 1000 cells were counted in each individual group. **P<0.01, ***P<0.001.

**Figure 3:**
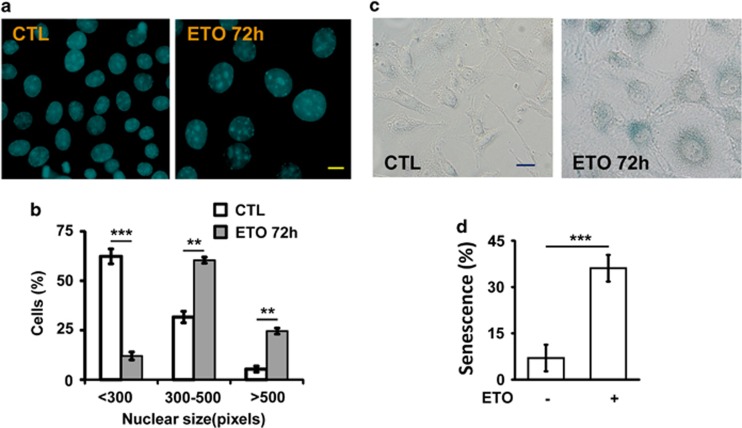
Long-term sublethal dose of ETO treatment induces genomic instability and cellular senescence. (**a** and **b**) Enlarged nuclei are observed in ETO-treated (10 μM, 72 h) adrenocortical Y1 tumor cells. (**a**) Examination of nuclear size by DAPI staining. The scale bar is 5 μm. (**b**) Quantitation of nuclear areas. The areas of nuclei from at least 100 Y1 cells in (**a**) were counted and compared in three independent experiments. (**c** and **d**) Cellular senescence are observed in ETO-treated adrenocortical Y1 tumor cells. (**c**) Cellular senescence is shown by beta-galatosidase activity after ETO treatment for 72 h. (**d**) Quantitation of cells with beta-galatosidase activity. These results are mean +/−s.d. from three independent experiments; more than 100 cells were counted in each individual group. Scale bar is 5 μM. **P<0.01, ***P<0.001.

Sublethal dose of ETO treatment did not induce cell death, then the cell cycle profile was further analyzed. In the presence of 10 μM ETO for 72 h, the population of cells in sub-G1 was not induced, whereas G1 and S phase were reduced dramatically (Figure 2a). In addition, the G2/M arrest was observed. To our surprise, the population of cells with polyploidy (>4N), the hallmark of genomic instability, was increased robustly. The ability of cells to enter the S phase was also confirmed by EdU incorporation assay. Consistent with our flow analysis, ETO treatment reduced EdU incorporation in both H295 and Y1 cells (Figure 2b). The M phase entry was also examined by mitotic index. Although G2/M population was increased in ETO-treated cells, the population of mitotic cells was reduced (Figure 2c). Thus, prolonged treatment of sublethal ETO-induced G2/M arrest and genomic instability. The genomic instability was also checked by measuring the nuclear size. We found that, during prolonged treatment of sublethal dose of ETO, the population of cells with larger nucleus was increased (Figures 3a and b). It has been shown that cells with genomic instability undergo cellular senescence if apoptosis is not triggered, thus we further checked the cellular senescence by acidic β-galatosidase activity assay. Under prolonged ETO treatment, ACT cells underwent senescence especially in those cells with an enlarged nucleus (Figures 3c and d). Thus, sublethal dose of ETO treatment induced genomic instability and cellular senescence.

### ETO induces centrosome amplification by activating cyclin A/CDK2, Chk2 and ERK1/2

**Figure 4:**
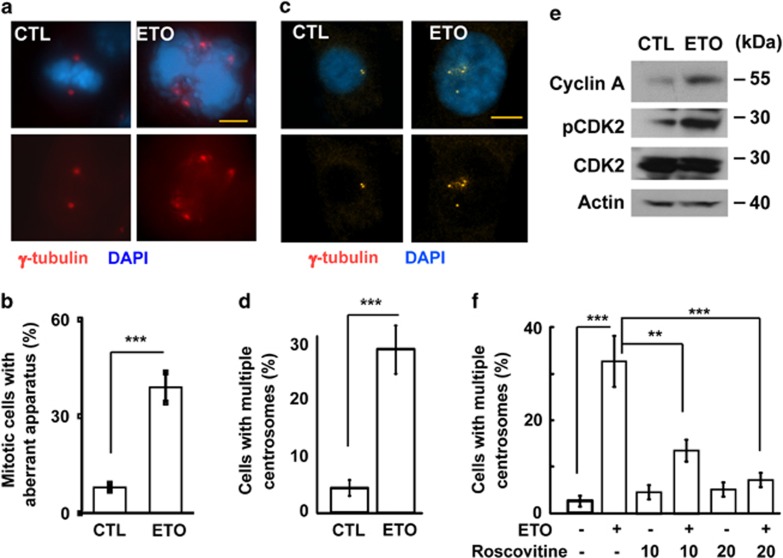
ETO treatment induces centrosome amplification in ACT cells. (**a**, **b**) ETO treatment (10 μM, 24 h) induces aberrant mitotic apparatus. (**a**) Immunofluorescence examination of mitotic cells by staining with DNA (DAPI, blue) and mitotic spindle poles (γ-tubulin, red) in Y1 cells. (**b**) Quantitation of mitotic Y1 cells with aberrant mitotic spindle poles. (**c**, **d**) ETO treatment induces centrosome amplification. (**c**) Immunostaining of centrosome with antibody against γ-tubulin. DNA is stained by DAPI. Scale bar is 5 μm. (**d**) Quantitation of Y1 cells with multiple centrosomes (>2 centrosomes) during interphase. (**e**) Upregulation of cyclin A and activation of CDK2 upon ETO treatment (10 μM, 24 h) in adrenocortical Y1 tumor cells. Extracts of ETO-treated Y1 cell lysates are analyzed by immunoblotting with antibodies against cyclin A, CDK2, phospho-CDK2 on Thr160, and actin. (**f**) Inactivation of CDK2 inhibits ETO-induced centrosome amplification in Y1 cells. Quantitation of Y1 cells containing multiple centrosomes in the presence or absence of ETO with or without CDK2 inhibitor, roscovitine. All results are expressed as the mean+/−s.d. from at least three independent experiments. **P<0.01, ***P<0.001.

We further investigated how ETO induced genomic instability. It has been shown that aberrant mitosis leads to genomic instability,^20^ and thus, we checked the mitotic apparatus upon ETO treatment. The population of cells that entered the M phase was reduced under the sublethal dose of ETO treatment, however, when those mitotic cells were checked, aberrant mitotic apparatus was observed (Figures 4a and b). Normally duplicated centrosomes were separated to the opposite sites of nucleus to ensure that the replicated chromosomes aligned in the equator plane (Figure 4a, left panel). In the presence of ETO, multiple mitotic spindle poles were observed and the chromosomes could not be aligned properly in the middle of the cells (Figure 4a, left panel). As multiple mitotic spindle poles arise from centrosome amplification, the centrosome copy number was examined during interphase. Normally, cells contain one (non-duplicated) or two (duplicated) centrosomes as shown by γ-tubulin staining. However, under ETO treatment, cells with more than two centrosomes (centrosome amplification) were observed (Figures 4c and d). Thus, the sublethal dose of ETO treatment induced centrosome amplification during interphase leading to aberrant mitotic apparatus at M phase.

The level of cyclin A is critical for cell cycle progression and centrosome duplication. Overexpression of cyclin A results in centriole splitting (the distance between the pair of centrioles larger than 2 μm) leading to supernumerary centrosomes.^21^ We further checked the expression of cyclin A and the activation of CDK2, because their aberrant upregulation induces centrosome amplification. The expression level of CDK2 was not affected, however, the abundance of cyclin A was increased and thus led to aberrant activation of CDK2 as shown by increased activating phosphorylation of CDK2 (Figure 4e). To further confirm the role of CDK2 in controlling ETO-induced centrosome amplification, different doses of CDK2 inhibitor, roscovitine, were co-treated with ETO in ACT cells. Inactivation of CDK2 alleviated the centrosome amplification in ETO-treated ACT cells in a dose-dependent manner (Figure 4f). Furthermore, CDK2 was depleted by siRNA and ETO-induced centrosome amplification was alleviated in CDK2-deficient ACT cells (Supplementary Figures S1A and B). Thus, CDK2 activity was required for sublethal dose of ETO treatment induced centrosome amplification.

**Figure 5:**
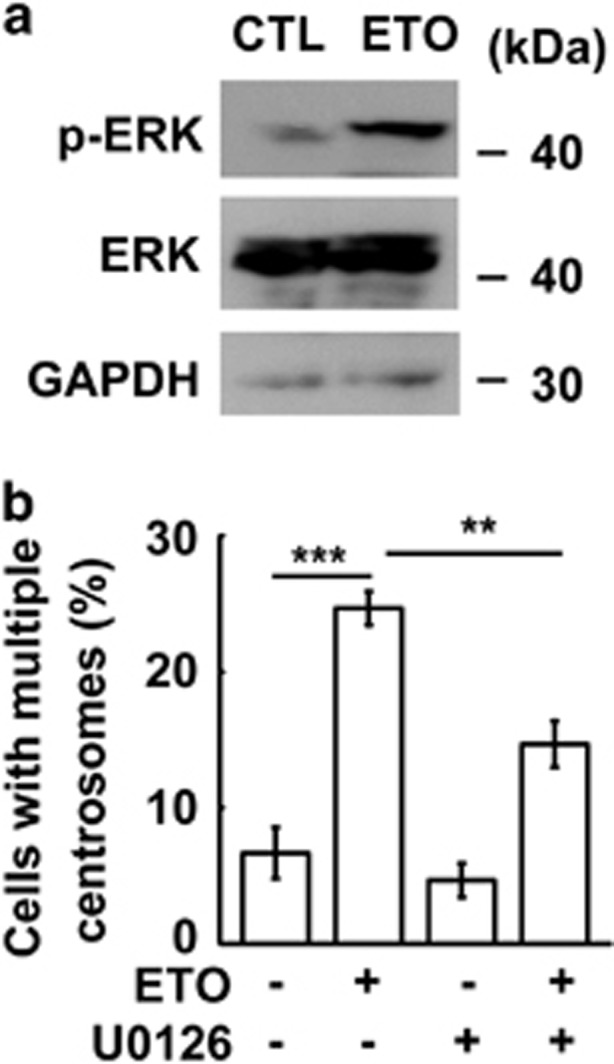
Inhibition of ERK1/2 inhibits ETO-induced centrosome amplification. (**a**) ERK1/2 is activated upon ETO treatment (10 μM, 24 h) in adrenocortical Y1 tumor cells. Extracts of ETO-treated Y1 cell lysates are analyzed by immunoblotting with antibodies against phospho-ERK1/2 on Thr202/Tyr204, ERK1/2 and GAPDH. (**b**) Inactivation of ERK inhibits ETO-induced centrosome amplification. Quantitation of Y1 cells containing multiple centrosomes in the presence or absence of ETO with ERK inhibitor, U0126. All results are expressed as the mean+/−s.d. from at least three independent experiments. **P<0.01, ***P<0.001.

It has been suggested that treatment of cells with ETO elicited Chk2 and ERK1/2 activation.^17, 39^ Activation of Chk2 and ERK1/2 also induces centrosome amplification.^17, 40^ Thus, we tested the involvement of Chk2 and ERK1/2 in controlling centrosome amplification of ETO-treated ACT cells. The levels of phosphorylated Chk2 and ERK were increased upon ETO treatment in ACT cells (Figure 5a and Supplementary Figure S2A) and inhibition of Chk2 and ERK by the specific inhibitors, Chk2 inhibitor II and U0126, respectively, reduced centrosome amplification, suggesting that ETO activated Chk2 and ERK signaling leading to centrosome amplification (Figure 5b and Supplementary Figure S2B). In addition, the expression of Chk2 was also depleted by siRNA and we found that ETO-induced centrosome amplification was reduced in Chk2-deficient ACT cells (Supplementary Figures S2C and D). Thus, ETO treatment activated cyclin A/CDK2, Chk2 and ERK1/2 followed by inducing centrosome amplification.

### Chloroquine alleviates DNA damage-induced centrosome amplification in ACT and osteosarcoma cells

**Figure 6:**
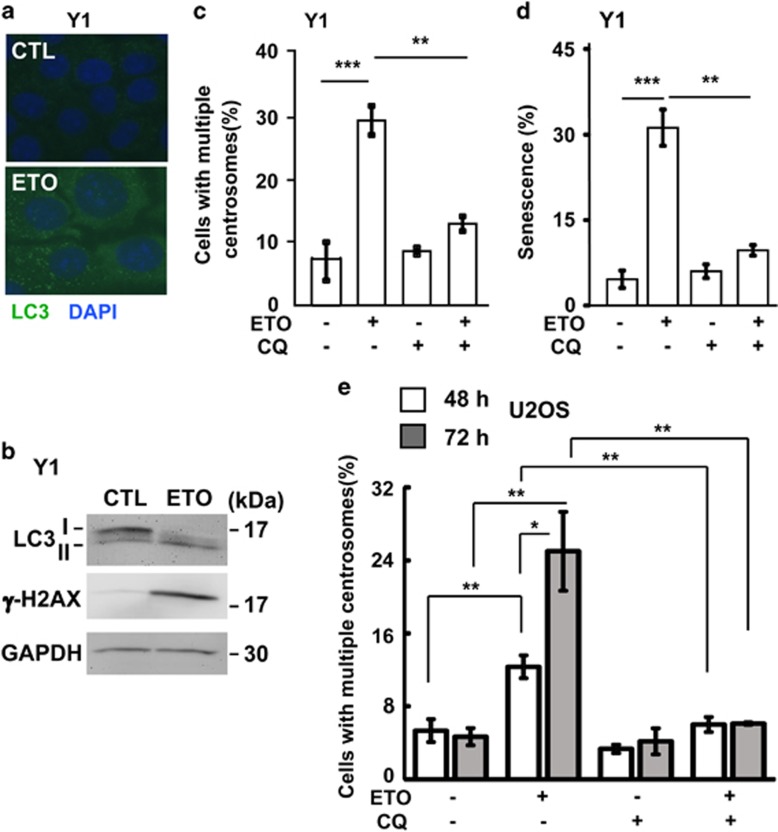
Chloroquine inhibits DNA damage-induced centrosome amplification. (**a**, **b**) ETO treatment (10 μM, 24 h) induces autophagy in adrenocortical Y1 tumor cells. (**a**) Immunostaining of autophagosomes with antibody against LC3 in the presence or absence of ETO. DNA is stained with DAPI. (**b**) Extracts of ETO-treated Y1 cell lysates are analyzed by immunoblotting with antibodies against LC3, γ-H2AX and GAPDH. (**c**, **d**) Chloroquine inhibits ETO-induced centrosome amplification (**c**) and senescence (**d**) in adrenocortical Y1 tumor cells. Quantitation of Y1 cells containing multiple centrosomes (**c**) or senescence (**d**) in the presence or absence of ETO with or without chloroquine. (**e**) Chloroquine inhibits ETO-induced centrosome amplification in osteosarcoma U2OS cells. Quantitation of U2OS cells containing multiple centrosomes in the presence or absence of ETO with or without chloroquine. All results are expressed as the mean+/−s.d. from at least three independent experiments. *P<0.05, **P<0.01, ***P<0.001.

**Figure 7:**
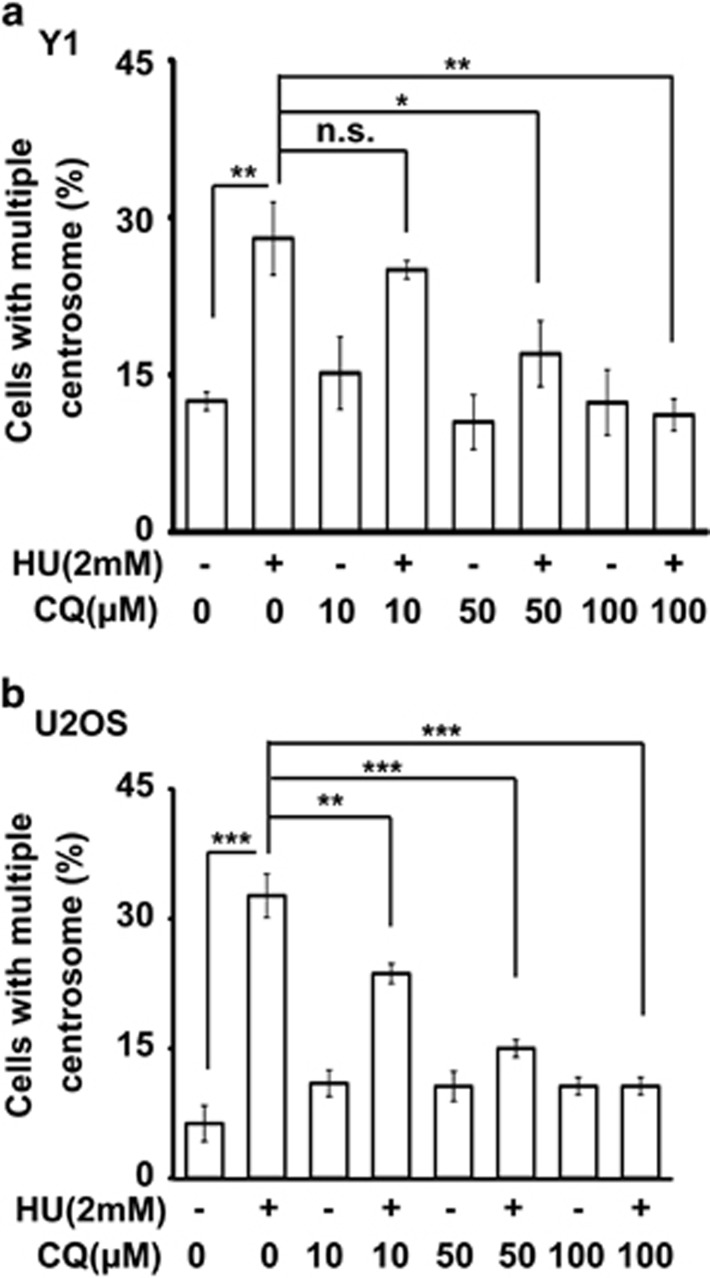
Chloroquine alleviates hydroxyurea-induced centrosome amplification. (**a**, **b**) Chloroquine inhibits hydorxyurea-induced (2 mM, 72 h) centrosome amplification in adrenocortical Y1 tumor (**a**) and osteosarcoma U2OS (**b**) cell lines. Quantitation of cells containing multiple centrosomes in the presence or absence of hydroxyurea with or without chloroquine. All results are expressed as the mean+/−s.d. from at least three independent experiments. n.s., no significance, *P<0.05, **P<0.01, ***P<0.001.

The role of autophagy in the maintenance of cellular homeostasis under stress condition is widely studied. However, it is still unclear whether autophagy participates in DNA damage-induced centrosome amplification. First, we checked whether ETO treatment activated autophagy and the autophagic puncta (LC3 puncta) was checked. In ACT cells, treatment with sublethal dose of ETO led to the accumulation of LC3 puncta as shown by immune-fluorescence staining (Figure 6a), and this was further confirmed by the increase of LC3-II in the ETO-treated lysate (Figure 6b), thus, ETO treatment indeed induced autophagy. Next, we checked whether autophagy participated in ETO-induced centrosome amplification by treating cells with the autophagy inhibitor, chloroquine. Treatment of cells with ETO induced centrosome amplification, and inhibition of autophagy by chloroquine inhibited ACT cell growth and reduced ETO-induced centrosome amplification (Supplementary Figures S3 and Figure 6c). Centrosome amplification induced cellular senescence in ETO-treated ACT cells (Figures 3c and d), and thus, we checked whether chloroquine alleviated cellular senescence. Indeed, treatment of chloroquine reduced ETO-induced cellular senescence (Figure 6d). Thus, treatment with ETO activated autophagy, in turn inducing centrosome amplification and leading to senescence. Next, we checked whether chloroquine inhibited centrosome amplification in other tumor cell lines. The osteosarcoma U2OS cell line, a model cell for studying centrosome homeostasis, was treated with ETO for 48 and 72 h. Treatment with ETO induced centrosome amplification in a time-dependent manner and this phenotype was inhibited by co-treatment with chloroquine (Figure 6e). In addition to ETO, it is known that treatment of hydroxyurea results in prolonged replication stress and induces centrosome amplification.^17^ We further checked whether hydroxyurea-induced centrosome amplification was inhibited by chloroquine. Hydroxyurea induced centrosome amplification in both ACT and osteosarcoma cell lines, and co-treatment of hydroxyurea with chloroquine inhibited centrosome amplification in a dose-dependent manner (Figures 7a and b). These data indicated that chloroquine inhibited DNA damage-induced centrosome amplification.

**Figure 8:**
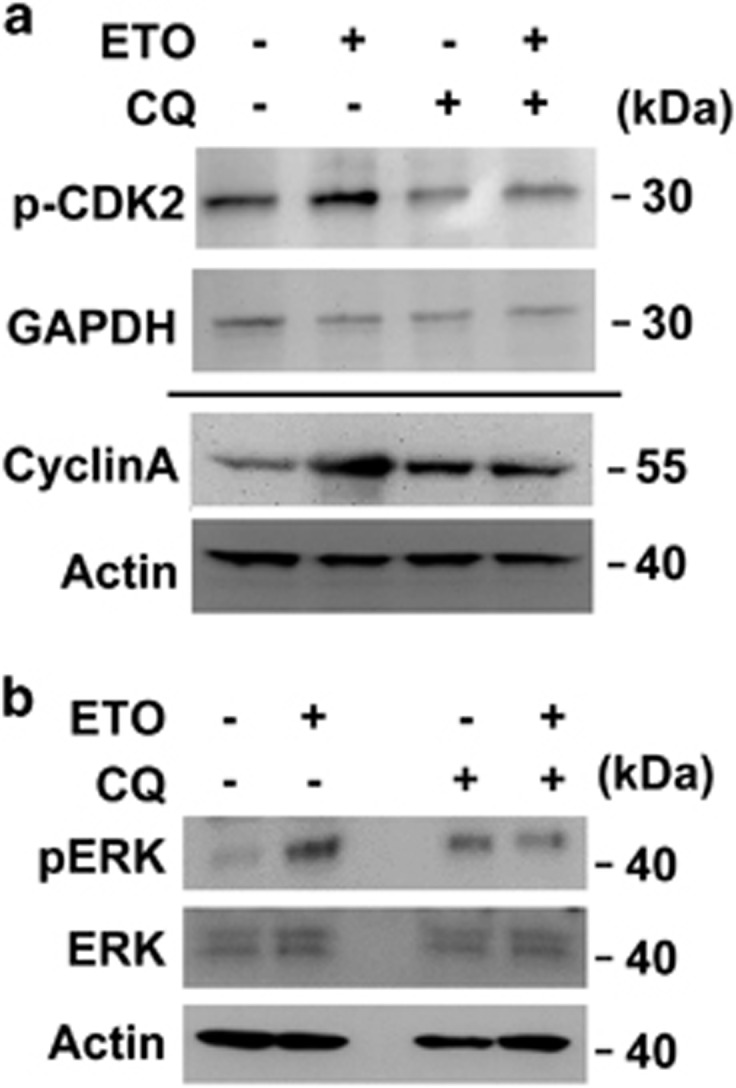
Chloroquine inhibits ETO-activated CDK2 and ERK1/2 in adrenocortical Y1 tumor cells. (**a**, **b**) Extracts of ETO-treated adrenocortical Y1 tumor cell lysates in the absence or presence of chloroquine (CQ) are analyzed by immunoblotting with antibodies against (**a**) cyclin A, actin, phospho-CDK2 on Thr160, GAPDH or (**b**) phospho-ERK on Thr202/Tyr204, ERK and actin.

We further checked whether chloroquine potentiated ETO-activated Chk2, CDK2 and ERK1/2. In ACT cells, chloroquine did not inhibit Chk2 activation (Supplementary Figure S2E) although inactivation of Chk2 reduced centrosome amplification indicating that the distinct role of DNA damage response in controlling centrosome homeostasis. In addition to transcriptional regulation, the abundance of cyclin A is also controlled by autophagy.^41^ Next, we checked whether the level of cyclin A was affected by chloroquine. Although chloroquine treatment induced the level of cyclin A slightly, double treatment of chloroquine and ETO blocked the upregulation of cyclin A. In addition, the total amount of CDK2 was not affected, but the phosphorylation of CDK2 was also inhibited by chloroquine in ETO-treated ACT cells (Figure 8a). We also checked the effect of chloroquine on ETO-activated ERK signaling. ETO treatment induced ERK1/2 activation and this was reversed by chloroquine treatment in ACT cells (Figure 8b). These data were consistent with our previous results that inactivation of CDK2 and ERK1/2 alleviated ETO-induced centrosome amplification.

## Discussion

In this study, we demonstrated that the sublethal dose of ETO treatment led to centrosome amplification in turn causing genomic instability. Genomic instability is the leading cause of malignancy for recurring tumor, thus it is important to uncover the molecular mechanism by which the sublethal dose of anti-neoplastic drugs induces centrosome amplification. Upon sublethal dose of ETO treatment, either DNA damage response or autophagy were activated and contributed to centrosome amplification in ACT cells (Supplementary Figures S4). It is well known that the DNA damage checkpoint kinase, Chk2, is important for the maintenance of cell cycle arrest and promotes DNA repair when cells suffer from DNA damage response.^42^ Here, we showed that the activation of Chk2 upon ETO treatment also induced centrosome amplification and contributed to genomic instability. In addition, we also found that autophagy had an important role in stress-induced centrosome amplification. Autophagy is a known protective mechanism that maintains cell survival during genomic stresses.^36^ Here, we showed that ETO-induced autophagy contributed to centrosome amplification and cellular senescence by activating ERK1/2 and cyclin A/CDK2, and these were reversed by treated with autophagy inhibitor, chloroquine.

It is straightforward to consider the autophagy inhibitor, chloroquine, as an enhancer to prompt the effect of anti-neoplastic drugs, such as ETO or hydroxyurea, in treating ACTs. The basal level of autophagy is required for tumor growth, in addition, the autophagic flux is upregulated to further sustain the tumor survival when these cancer cells suffer from sublethal dose of antitumor drugs.^27^ These tumor cells are more susceptible to cell death when autophagy is inhibited. Growing evidences support that chloroquine is a potent antitumor drug in treating several tumors, such as liver and blood cancers.^33, 35^ In our current study, we found that treatment of ACT cells with chloroquine also inhibited cell growth. In addition, chloroquine also inhibited centrosome amplification in the ETO-treated cells. Centrosome amplification facilitates recurring tumor,^22^ and here, we showed that chloroquine inhibited DNA damage-induced centrosome amplification. Thus, chloroquine could be a new candidate of combined chemotherapy in treating with ACT. It can facilitate tumor cell death and also prevent centrosome amplification in the remaining cells, and thus, avoid malignancy when chemotherapy is stopped in patients.

ERK1/2 signaling promotes cell proliferation in response to extracellular signaling, in addition, it is activated by ETO and leads to chemoresistance in the tumor.^43^ It is known that supernumerary centrosomes contribute to chemoresistance and poor outcome in tumor cells.^44^ Here, we showed that ETO treatment activated ERK1/2, followed by centrosome amplification in ACT cells. This ETO-induced ERK activation was facilitated by autophagy as inhibition of autophagy alleviated ERK activation and centrosome amplification. The connection between ERK signaling and autophagy is not well studied. Depletion of autophagy-related proteins, such as Atg5 and Atg7, reduces ERK phosphorylation.^45^ On the contrary, inactivation of MEK/ERK signaling inhibits autophagy in response to chemotherapy drugs.^46^ It seems that the autophagic machinery is required for the activation of ERK signaling; the activated ERK can further promote autophagic flux upon chemotherapy. Here, we showed that chloroquine inhibited ETO-induced ERK activation. The possible mechanism might be that chloroquine blocked the autophagic flux, and thus, disturbed the homeostasis of autophagic machinery leading to inhibition of ERK signaling; this hypothesis still needs to be tested.

In summary, here, we uncovered the novel function of chloroquine in preventing centrosome amplification, thus preventing genomic instability. In our study, chloroquine inhibited DNA damage-induced centrosome amplification in both ACT and osteosarcoma cell lines. As poor outcome of patients suffer from these tumors after chemotherapy, our study showed the possible mechanism of recurring tumor due to autophagy-induced centrosome amplification.

## Materials and methods

### Cell culture and drug treatment

Human adrenocortical H295 and mouse adrenocortical Y1 cell lines were grown in Dulbecco's modified Eagle medium-F12 medium supplemented with 10% fetal bovine serum at 37 °C in a humidified atmosphere at 5% CO_2_. These cells were regularly examined for the presence of SF-1 and mycoplasma contamination by immunoblot, immunofluorescence and DAPI staining according to the guidelines. Human osteosarcoma U2OS cell line was grown in Dulbecco's modified Eagle medium and 10% fetal bovine serum at 37 °C in a humidified atmosphere at 5% CO_2_. For drug treatment, cells were incubated with or without 10 or 20 μM roscovitine (Merck, Darmstadt, Germany), 2 mM hydroxyurea, 10, 50 or 100 μM chloroquine, or 10 μM Chk2 Inhibitor II (Sigma, St. Louis, MO, USA) for 24 h before analysis. For induction of DNA damage response, cells were treated with 1, 10 or 100 μM ETO for different time points.

### Antibodies

The following antibodies were obtained commercially: anti-γ-tubulin (T6557), anti-Cyclin A (C4710) and anti-α-tubulin (T9026) (Sigma), anti-CDK2 (#2546), anti-Chk2 (#6334), anti-Chk2 phospho-Thr68 (#2661), anti-ERK1/2 (#4695), anti-phospho-ERK1/2 on Thr202/Tyr204 (#4370), anti-Cyclin E (#4132), anti-CDK2 phospho-Thr160 (#2561) and anti-LC3A/B (#12741) (Cell Signaling, Beverly, MA, USA), anti-H2AX phospho-Ser139 (ab2893, Abcam, Cambridge, UK), anti-actin (GTX109639) and anti-GAPDH (GTX100118) (Genetex, Irvine, CA, USA).

### Immunofluorescence microscopy

Cells were grown on glass cover slips at 37 °C before fixation with ice-cold methanol at −20 °C for 6 min. After blocking with 5% bovine serum albumin for 1 h, cells were incubated with antibodies for 24 h at 4 °C. After extensive washing with phosphate-buffered saline (PBS), cells were incubated with fluorescein isothiocyanate-conjugated and Cy3-conjugated secondary antibodies (Invitrogen, Carlsbad, CA, USA) for 1 h in the dark. The nuclei were stained with 4′, 6-diamino-2-phenylindole (DAPI, 0.1 μg/ml) simultaneously. After extensive washing, the cover slips were mounted in 50% glycerol on glass slides. Fluorescent cells were examined with an AxioImager M2 fluorescence microscope (Zeiss, Feldbach, Switzerland). The number of centrosomes from more than 100 cells was counted under the microscope in three independent experiments and shown as mean±s.d. Student's *t*-test was performed to analyze the difference between different groups.

### MTT assay

Cells were washed with PBS followed by addition of 1 ml MTT solution (2 mg/ml in PBS) in each well. After incubation for 3 h at 37 °C, 2 ml DMSO was added and incubation in the dark for additional 30 min. Absorbance was measured at the wavelength of 570 nm.

### Cell growth assay and western blotting analysis

Cells were trypsinized and resuspended in PBS for cell number counting or centrifuged for further western blotting analysis following drug treatment. Centrifuged cells were further lysed with lysis buffer containing 0.5% NP-40, 300 mM NaCl, 1 mM EDTA and the protease inhibitor cocktail (Roche, Mannheim, Germany) followed by centrifugation (15 000 r.p.m., 4 °C). Supernatant was collected and further analyzed by western blotting.

### RNA interference

CDK2 of adrenocortical Y1 cells was depleted using annealed siRNA with the target sequence: siCDK2: 5'-gguguacccaguacugcca [dt] [dt]-3'.^47^ Scrambled siRNA with the target sequence: 5'-gaucauacgugcgaucaga [dt] [dt]-3' was purchased from Sigma.

siRNA against Chk2 was purchased from Ambion (s78549, silencer select pre-designed siRNA, Thermo Fisher Scientific, Waltham, MA, USA).

For siRNA transfection, 10 μl of Lipofectamine 2000 (Invitrogen) were mixed first with 500 μl Opti-MEM medium (Life Technologies, Grand Island, NY, USA) for 5 min, then with 2 μl siRNA (100 μM) in 500 μl Opti-MEM medium, incubated at room temperature for 20 min before the mixture was layered onto cells in 1 ml Dulbecco's modified Eagle medium/F12 (100 nM working concentration). Cells were harvested for immunoflurorescence and immunoblotting 72 h after transfection.

### Cell cycle analysis

The cell cycle profile was analyzed by fluorescence-activated cell sorting according to a published method.^48^ Briefly, cells were collected by trypsinization and re-suspended with PBS. Following centrifugation at 1000 r.p.m. for 5 min, cells were re-suspended with PBS-E (1 mM EDTA in PBS). After centrifugation, the pellet was re-suspended with 0.5 ml PBS-E and fixed with ice-cold 70% ethanol overnight at 4 °C. Fixed cells were washed with PBS-E and stained with propidium iodide (Southern Biotech, Birmingham, AL, USA) at room temperature for 1 h. DNA content of PI-stained cells was measured by FACScan (Becton-Dickinson, San Diego, CA, USA) and further analyzed by Kaluza software (Beckman Coulter, Brea, CA, USA).

### Senescence assay and EdU incorporation assay

Senescent cells were stained by detecting senescence-associated β-galactosidase activity according to the manufacturer's instruction (Cell Signaling, Beverly, MA, USA). For EdU incorporation assay, EdU-positive cells were stained by detecting fluorescence EdU signaling according to the manufacturer's instruction (Invitrogen). Cells were co-stained with DAPI to visualize nuclei.

### Statistical analysis

All results are expressed as the mean+/−s.d. from at least three independent experiments; more than 100 cells were counted in each individual group. Student's *t*-test was performed to determine statistical significant in two experimental comparisons.
