# Supplementary material for: Chloroquine alleviates etoposide-induced centrosome amplification by inhibiting CDK2 in adrenocortical tumor cells
Source: Oncogenesis. 2015 Dec 21;4(12):e180–. doi: 10.1038/oncsis.2015.37 (PMC4688395; doi:10.1038/oncsis.2015.37)
Supplement: Supplementary Information [file oncsis201537x1.doc]

**Supplementary figures**

**Supplementary figure 1.**

**
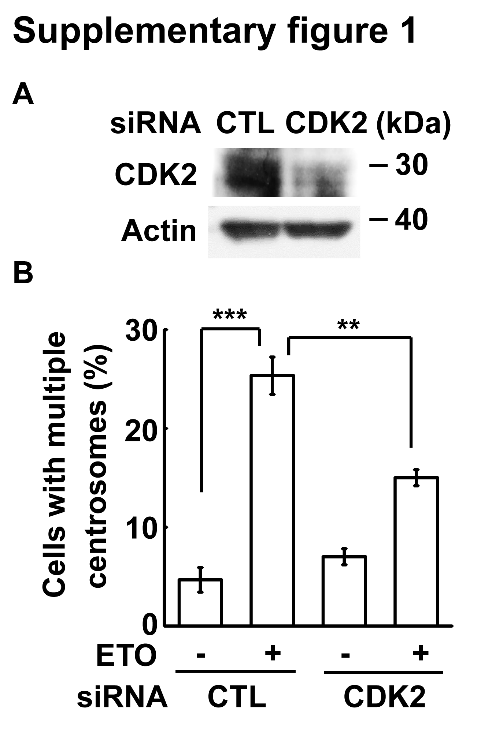
**

**Supplementary figure 1. Depletion of CDK2 inhibits ETO-induced centrosome amplification in adrenocortical Y1 tumor cells.**

1. CDK2 is depleted by specific siRNA. Extracts of siCTL (control) or siCDK2 transfected Y1 cells were analyzed by immunoblotting with antibodies against CDK2 and actin. (B) Depletion of CDK2 reduces centrosome amplification in ETO-treated adrenocortical Y1 tumor cells. Quantitation of cells with multiple centrosomes in siCDK2 transfected Y1 cells in the presence or absence of ETO. These results are mean +/- SD from three independent experiments; more than 300 cells were counted in each individual group. **: p<0.01, ***: p<0.001.

**Supplementary figure 2.**


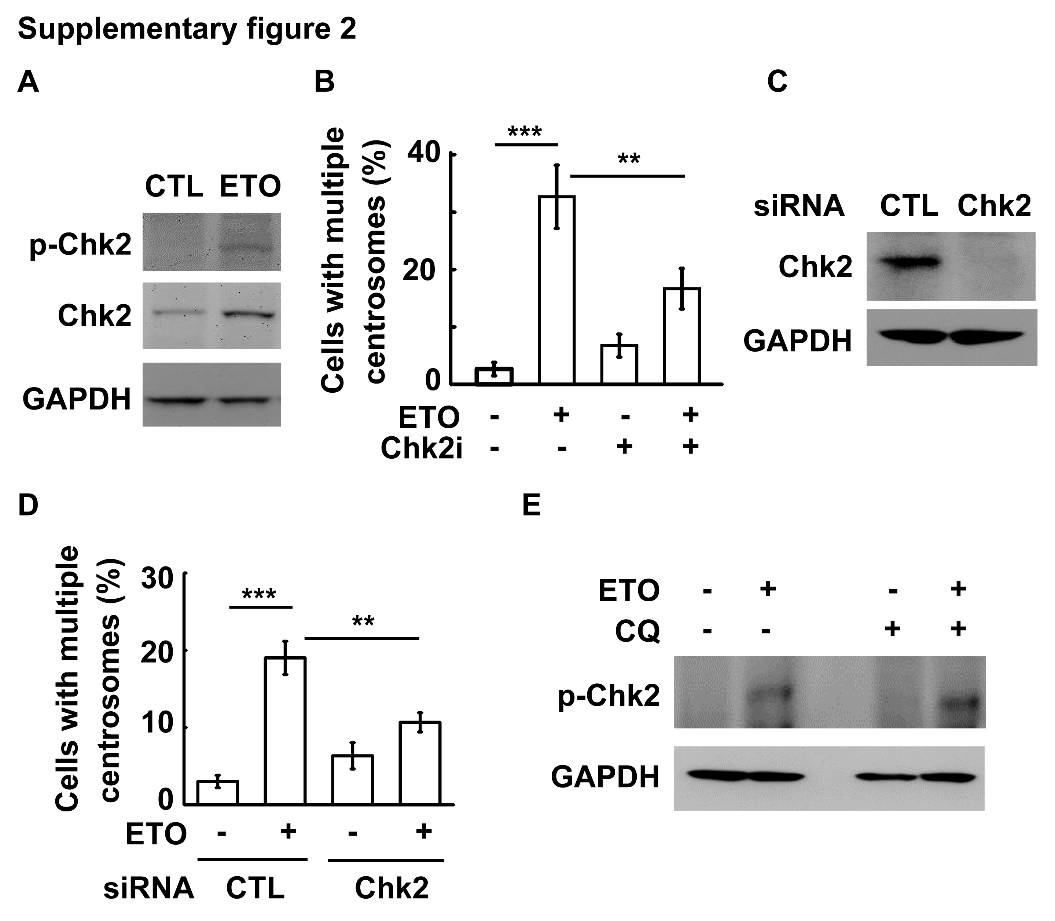


**Supplementary figure 2. Inactivation of Chk2 inhibits ETO-induced centrosome amplification in adrenocortical tumor cells.**

(A) ETO activates Chk2. Extracts of ETO-treated cells are analyzed by immunoblotting with antibodies against Chk2, phospho-Chk2 on Thr68, and GAPDH. (B) Inactivation of Chk2 reduces centrosome amplification in ETO-treated adrenocortical tumor cells. Quantitation of cells with multiple centrosomes in the presence or absence of Chk2 inhibitor II (Chk2i). (C) Chk2 is depleted by specific siRNA. Extracts of siCTL (control) or siChk2 transfected Y1 cells were analyzed by immunoblotting with antibodies against Chk2 and GAPDH. (D) Depletion of Chk2 reduces centrosome amplification in ETO-treated adrenocortical Y1 tumor cells. Quantitation of cells with multiple centrosomes in siChk2 transfected Y1 cells in the presence or absence of ETO. These results are mean +/- SD from three independent experiments; more than 300 cells were counted in each individual group. **: p<0.01, ***: p<0.001. (E) Chloroquine does not inhibit Chk2 activation. Extracts of ETO-treated cells in the presence or absence of chloroquine are analyzed by immunoblotting with antibodies against phospho-Chk2 on Thr68 and GAPDH.

**Supplementary figure 3.**


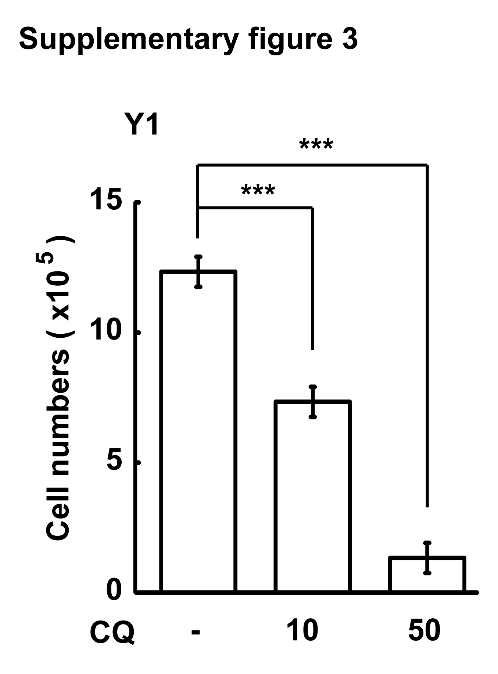


**Supplementary figure 3. Chloroquine reduces adrenocortical tumor cell growth.**

The numbers of adrenocortical tumor Y1 cells are quantified in the presence of chloroquine (CQ) at different concentrations. **: p<0.01, ***: p<0.001.

**Supplementary figure 4.**


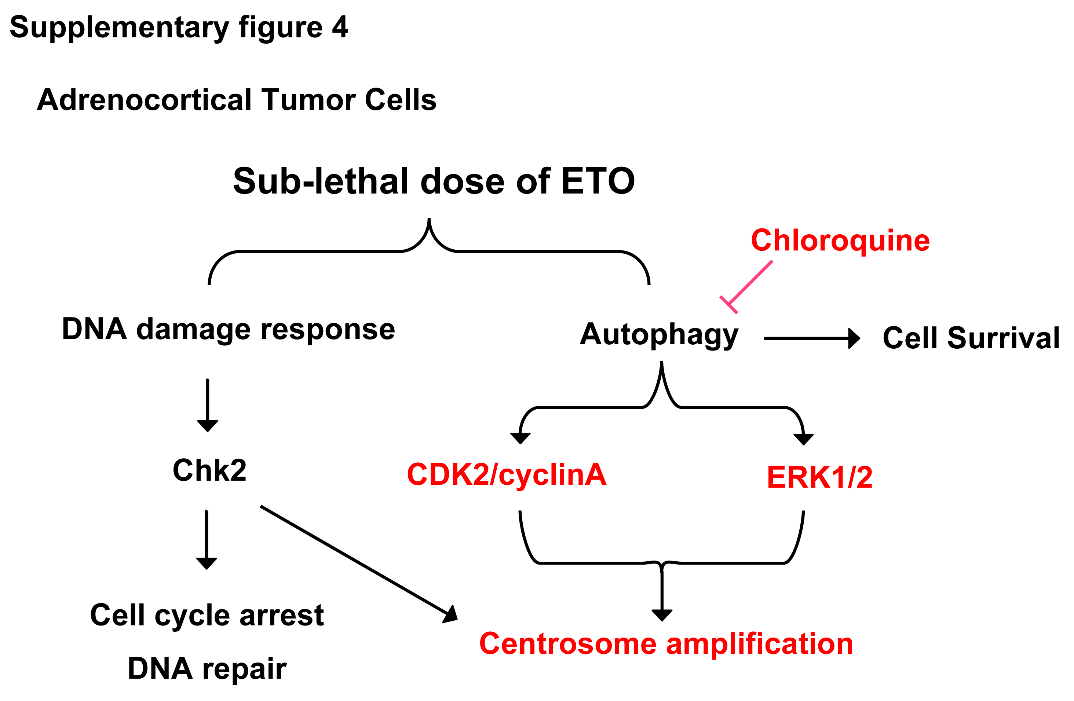


**Supplementary figure 4. A flow chart depicting chloroquine action that prevents sub-lethal dose of ETO-induced centrosome amplification.**

In adrenocortical tumor cells, sub-lethal dose of ETO treatment activates DNA damage response and autophagy. DNA damage response activates Chk2, which is independent of autophagic flux, thus induces centrosome amplification. ETO-induced autophagy maintains cell survival and induces centrosome amplification. Chloroquine reduces ACT cell growth and also blocks centrosome amplification by inhibiting ERK1/2 and CDK2 signaling.
